# NextPolish2: A Repeat-aware Polishing Tool for Genomes Assembled Using HiFi Long Reads

**DOI:** 10.1093/gpbjnl/qzad009

**Published:** 2024-01-04

**Authors:** Jiang Hu, Zhuo Wang, Fan Liang, Shan-Lin Liu, Kai Ye, De-Peng Wang

**Affiliations:** School of Automation Science and Engineering, Faculty of Electronic and Information Engineering, Xi’an Jiaotong University, Xi’an 710049, China; GrandOmics Biosciences, Beijing 102206, China; GrandOmics Biosciences, Beijing 102206, China; GrandOmics Biosciences, Beijing 102206, China; Department of Entomology, College of Plant Protection, China Agricultural University, Beijing 100193, China; School of Automation Science and Engineering, Faculty of Electronic and Information Engineering, Xi’an Jiaotong University, Xi’an 710049, China; GrandOmics Biosciences, Beijing 102206, China

**Keywords:** Telomere-to-telomere, Genome assembly, Genome polishing, Error correction, HiFi long read

## Abstract

The high-fidelity (HiFi) long-read sequencing technology developed by PacBio has greatly improved the base-level accuracy of genome assemblies. However, these assemblies still contain base-level errors, particularly within the error-prone regions of HiFi long reads. Existing genome polishing tools usually introduce overcorrections and haplotype switch errors when correcting errors in genomes assembled from HiFi long reads. Here, we describe an upgraded genome polishing tool — NextPolish2, which can fix base errors remaining in those “highly accurate” genomes assembled from HiFi long reads without introducing excessive overcorrections and haplotype switch errors. We believe that NextPolish2 has a great significance to further improve the accuracy of telomere-to-telomere (T2T) genomes. NextPolish2 is freely available at https://github.com/Nextomics/NextPolish2.

## Introduction

Complete and accurate genomes provide fundamental tools for scientists to capture a full spectrum of genomic variants and use such information to understand the evolutionary basis of various diseases and other biological phenotypes [1]. Hence, a complete and gapless genome, also known as a telomere-to-telomere (T2T) genome, has been emerging as a new hotspot in the field of genomics [2–8]. Typically, we obtain a T2T genome with datasets including both high-accuracy PacBio high-fidelity (HiFi) long reads and Oxford Nanopore Technologies (ONT) error-prone ultra-long reads [2]. Compared to the genomes generated using noisy long reads, genomes obtained using HiFi long reads have considerably higher quality — far fewer errors at the level of single nucleotides and small insertions and deletions (InDels) [9,10]. However, they still contain a handful of assembly errors in chromosomal regions where HiFi long reads stumble as well, such as homopolymer or low-complexity microsatellite regions (Figures S1 and S2). Additionally, a typical gap-filling step is accomplished using ONT ultra-long reads which contain a certain amount of errors that need to be corrected [11]. Hence, the current T2T genomes assembled using the cutting-edge sequencing platforms still require further improvement in terms of consensus accuracy. For example, the Human Genome T2T Consortium has applied multiple tools and extensive manual validation to increase the assembly quality value (QV) from 70.2 to 73.9 for the T2T assembly of a human genome (CHM13) [11].

Error correction for a T2T genome assembly is challenging because (1) complex segmental duplications and large tandem repeats, such as centromeric satellite arrays, could potentially induce overcorrections or false negative corrections; (2) local haplotype needs to be maintained; and (3) there are technology-specific biases for different sequencing platforms [12]. Therefore, although there are many state-of-the-art polishing tools available, such as Pilon [13], Racon [14], and NextPolish [15], these tools were designed for genomes assembled from noisy long reads and mainly aimed at errors at levels of single nucleotides and small InDels. They can hardly handle assembly errors sourced from HiFi long reads, without taking the aforementioned challenges into consideration.

Here, we present an upgraded genome polishing tool, NextPolish2, for error correction of T2T genomes constructed mainly using HiFi long reads. Compared to the up-to-date polishing pipeline Racon + Merfin (RM) [11], adopted to polish the human T2T genome assembly of CHM13, NextPolish2 can fix base errors in “highly accurate” draft assemblies without introducing overcorrections, even in regions with highly repetitive elements. Through the built-in phasing module, it can not only correct the error bases, but also maintain the original haplotype consistency. In fact, our evaluation shows that it even slightly reduces switch errors in heterozygous regions.

## Method

### Algorithm

**Figure 1:**
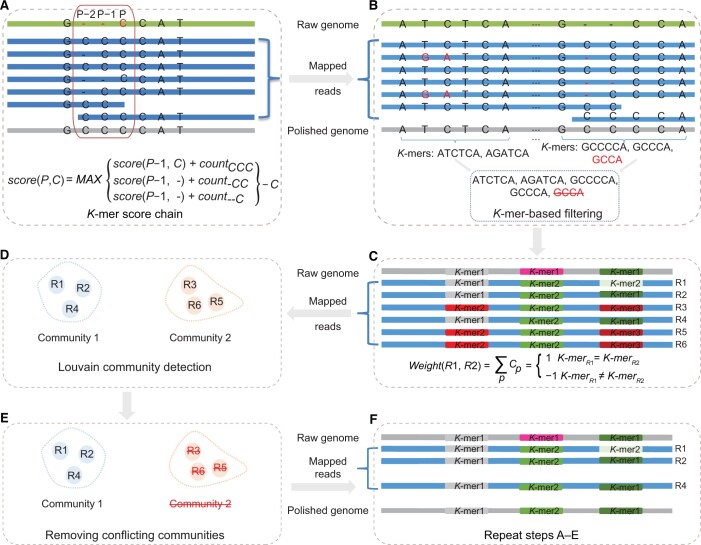
NextPolish2 pipeline **A**. The schematic of the *K*-mer score chain algorithm. The score of the base “C” at position P is that the maximum of the scores of its predecessor bases (C/deletion) at position P−1 plus the counts of their corresponding 3-mers (CCC, -CC, and --C), and then minus the valid depth (6) of position P. **B**. Extracting and filtering *K*-mers at LQRs using the *K*-mer datasets. **C**. Calculating weights between reads using the count of *K*-mers. **D**. Grouping reads using the Louvain community detection algorithm. **E**. Using reads from only one community for subsequent analyses and discarding reads from other communities. **F**. Repeating steps (A–E) until there are no conflicting communities. LQR, low-quality region; R, read.

NextPolish2 followed the *K*-mer score chain (KSC) algorithm of its previous version to perform an initial rough correction [15], and detected low-quality positions (LQPs) where the chosen alleles account for ≤ 95% of the total during a traceback procedure (**Figure 1**A). Next, it merged the adjacent LQPs into low-quality regions (LQRs), and then for each LQR it extracted *K*-mers from the HiFi long reads that can map across those LQRs. The *K*-mer set of each LQR was subsequently filtered using *K*-mer datasets generated from high-quality short reads (Figure 1B). Afterwards, it defined *K*-mer sets that contain ≥ 2 valid *K*-mers as heterozygous and used them to calculate weights between reads spanning the same LQRs. It then applied the Louvain community detection algorithm [16] to group reads from the same haplotype or repeat copy. We defined two conflict communities (*C*_1_,* C*_2_) as *weight*(*C*_1_, *C*_2_) < 0, which are located in the same region but from different haplotypes or repeat copies (Figure 1C and D). For the conflict communities, as a default setting, we kept the community that shares the most *K*-mers with the reference and removed reads from other communities (Figure 1E), which helps to maintain the same haplotype as the reference sequence. Users can also keep the largest community of their own preference. We repeated the aforementioned procedure until all conflict communities were resolved (the number of iterations can be adjusted according to user settings, Figure 1F), and then used the KSC algorithm to generate a draft consensus sequence. The draft consensus sequences may still contain a small number of LQRs. For those LQRs not spanned by any valid *K*-mers, we used the *K*-mer from the reference sequence as the correct *K*-mer to avoid overcorrection. For LQRs spanned by multiple valid *K*-mers, the *K*-mer with the highest number was defined as the correct one. Finally, we updated the draft consensus sequence using these correct *K*-mers and generated the final consensus sequence.

### Performance evaluation

We evaluated the performance of NextPolish2 against RM using four datasets, including (1) HiFi long reads and Illumina short reads simulated based on a simulated highly heterozygous diploid *Arabidopsis thaliana* genome; (2) published sequencing data of *A*. *thaliana* (Col-XJTU); (3) *Homo sapiens* (HG002); and (4) *Homo sapiens* (CHM13) (Table S1). We first applied hifiasm (v0.18.5) [10] to obtain a genome assembly for each dataset. In addition to a primary assembly (a complete assembly with long stretches of phased blocks), we generated two haplotype-resolved assemblies (two complete assemblies consisting of haplotigs, representing an entire diploid genome) for the HG002 genome with the available trio binning dataset. All the assemblies (primary and haplotype-resolved assemblies) reached QV scores of *ca.* 48–60 (**Table 1**). Then, we mapped HiFi long reads of each sample onto their corresponding genome assemblies using Winnowmap2 (v2.03) [17], a repeat-aware alignment tool that was adopted in the RM polishing pipeline. After that, we applied both Nextpolish2 and RM pipeline to conduct error correction separately for each genome assembly. Finally, we applied yak (v0.1; https://github.com/lh3/yak) for the simulated datasets and Merqury (v1.3) for the actual biological datasets [18] to evaluate QV scores and switch errors of the reference genomes and the polished genomes using *K*-mers from Illumina reads. Additionally, we applied meryl (v1.3; https://github.com/marbl/meryl) to detect *K*-mer changes between the reference genomes and the polished genomes to evaluate the challenge of overcorrection.

**Table 1:** Statistics of genome polishing results

Source	Software	QV	Switch error rate (‰)	Changed *K*-mers	Potential overcorrection *K*-mers	Wall clock time (min)	Maximum RAM (GB)
*A. thaliana* (simulated data, primary contigs)	hifiasm (primary)	47.67	1.99				
	Racon	43.12	765.95	6,384,788	89,878	20.77	17.01
	NextPolish	45.01	1058.34	6,159,005	36,758	**2.00**	**2.72**
	RM	52.18	737.45	6,207,453	114	26.73	17.01
	NextPolish2	**65.42**	**0.35**	**25,869**	**0**	2.53	34.34
*A. thaliana* (Col-XJTU, primary contigs)	hifiasm (primary)	58.03					
	Racon	46.58		53,462	213,624	14.33	17.25
	NextPolish	57.21		69,606	3134	**1.07**	**2.19**
	RM	63.89		7220	48	13.88	15.86
	NextPolish2	**64.26**		**1477**	**0**	6.28	8.03
*H. sapiens* (HG002, primary contigs)	hifiasm (primary)	60.25	0.15				
	RM	**63.52**	5.40	17,835,299	2895	544.64	206.07
	NextPolish2	62.87	**0.14**	**32,508**	**45**	**88.72**	**74.25**
*H. sapiens* (HG002, paternal contigs)	hifiasm (trio)	59.77	0.21				
	RM	63.44	0.69	3,711,007	1889	297.92	138.59
	NextPolish2	**63.49**	**0.20**	**588,415**	**316**	**92.65**	**50.26**
*H. sapiens* (HG002, maternal contigs)	hifiasm (trio)	59.78	0.33				
	RM	63.23	1.60	4,940,002	2088	320.21	154.30
	NextPolish2	**63.29**	**0.30**	**403,062**	**183**	**109.05**	**51.90**
*H. sapiens* (CHM13, primary contigs)	hifiasm (primary)	63.78/41.25					
	RM	**68.18/**39.83		417,723	1245	435.49	181.08
	NextPolish2	67.45/**41.87**		**13,061**	**26**	**88.43**	**73.26**

*Note*: The switch error rates of the Col-XJTU and CHM13 assemblies were unable to be evaluated due to missing parental sequencing dataset. The QV of the CHM13 assembly was evaluated by Merqury using short reads (left) and Minimap2 using the CHM13 T2T genome (right), and the QV on the right may be underestimated due to inconsistencies between assemblies and reference sequences and inaccurate alignments in some regions. Only the time for genome correction was counted, and the time for read mapping was not included. hifiasm (primary) refers to the primary hifiasm assembly, and hifiasm (trio) refers to the haplotype-resolved hifiasm assembly with trio binning. The best value for each metrics is indicated in bold. The *A. thaliana* datasets were run on the computer with 5 CPUs and 128 GB RAM of memory, and the *H. sapiens* datasets were run on the computer with 5 CPUs and 256 GB RAM of memory. RM, Racon + Merfin; QV, quality value; RAM, random access memory; T2T, telomere-to-telomere; CPU, central processing unit; GB, gigabyte; *A*. *thaliana*, *Arabidopsis thaliana*; *H*. *sapiens*, *Homo sapiens*.

## Results

### NextPolish2 improves QV without introducing switch errors

Regarding the *A*. *thaliana* genome, NextPolish2 improved QV from 47.67 to 65.42 for the simulated dataset and 58.03 to 64.26 for the Col-XJTU dataset, higher than that of RM (52.18 and 63.89, respectively). More importantly, NextPolish2 reduced the switch error rate of the simulated genome from 1.99‰ to 0.35‰; however, RM increased it to 737.45‰, suggesting that RM introduces a lot of haplotype switch errors (Table 1). For the human genome, the QV scores of the two software were evenly matched when working on the HG002 haplotype-resolved assemblies (63.49 by NextPolish2 and 63.44 by RM for the paternal assembly; 63.29 by NextPolish2 and 63.23 by RM for the maternal assembly); when working on the primary assembly, RM produced marginally higher QV scores (63.52 for HG002 and 68.18 for CHM13) than NextPolish2 (62.87 for HG002 and 67.45 for CHM13). However, the QV advantage of RM came at the cost of overcorrections and breaking haplotype blocks. For example, QV estimation based on reference alignment showed a decrease from 41.25 to 39.83 of RM but an increase from 41.25 to 41.87 of NextPolish2, suggesting that RM introduces overcorrections (see “NextPolish2 rarely introduces overcorrections” section for more details). Besides, the polished assemblies by RM contained more switch errors (5.40‰, 0.69‰, and 1.60‰ on the child, paternal, and maternal assemblies, respectively) than those of the original assemblies (0.15‰, 0.21‰, and 0.33‰, respectively) and the polished assemblies by NextPolish2 (0.14‰, 0.20‰, and 0.30‰, respectively) for all HG002 datasets. We assume that the RM pipeline may not design any particular modules to deal with haplotype switch errors, as it was developed to correct the CHM13 genome assembly that is a homozygous cell line based and thus contains a limited number of heterozygous loci.

### NextPolish2 rarely introduces overcorrections

We calculated two metrics: “changed *K*-mers” and “potential overcorrection *K*-mers” to evaluate the overcorrection issue. The former is the count of *K*-mers that are present in a polished assembly but not in its reference genome, whilst the latter is the count of *K*-mers that are present in a polished assembly but neither in its reference genome nor in Illumina short reads of the same sample. If a polishing tool introduces too many overcorrections, the polished assembly will contain lots of “changed *K*-mers” and “potential overcorrection *K*-mers”, because introducing a new *K*-mer with a length of *k* may indirectly introduce $\leq 2\times \left(k-1\right)$  *K*-mers overlapping with this new *K*-mer. In the case that a polishing tool introduces numerous haplotype switch errors, the polished assembly will consist of many “changed *K*-mers”, but without an increase of “potential overcorrection *K*-mers”, as the introduced heterozygous *K*-mers exist in the Illumina short reads. Compared with NextPolish2 on the human datasets, RM introduced ∼ 5.31–547.64 times more “changed *K*-mers” and ∼ 4.98–63.33 times more “potential overcorrection *K*-mers” without any significant improvement of QV. This suggests that RM overcorrects many authentic base sequences on the reference genomes by breaking haplotype blocks, resulting in a higher switch error (Table 1). We further tested NextPolish2 and RM on three additional datasets (Table S2) and counted the potential homozygous *K*-mer changes (Table S3), showing that RM is more likely to introduce overcorrections than NextPolish2.

Error correction of repetitive regions is a challenge and can be easily overcorrected because of their high similarity. To verify the overcorrection estimation, we downloaded 158 sequences of transposable elements (TEs) identified in the Col-CEN genome assembly from The Arabidopsis Information Resource (TAIR, https://www.arabidopsis.org/) — the source genome that was used to generate the simulated data of *A*. *thaliana*. Those TEs were used to evaluate the error correction performance for those highly repetitive regions of the genome. By comparing the mapping identity rate between the polished TEs and their corresponding references using Minimap2 (v2.24, -cx asm10) [19], we found that a total of 149 TEs were successfully assembled. However, only 68.46% of them could map to their corresponding TE references with an identity rate of 100%. After genome polishing, NextPolish2 increased the ratio from 68.46% to 91.95% and no TE was introduced overcorrection, but RM decreased the ratio from 68.46% to 12.75%, and about 80.54% of TEs had a lower identity after genome polishing (Table S4).

On the other hand, because the CHM13 T2T genome is carefully corrected and manually verified [11], we applied it as a reference sequence to evaluate the actual error correction performance on the CHM13 assembly. To avoid inconsistencies between the assembly and the reference sequence as well as inaccurate alignments as much as possible, we split the CHM13 T2T genome into 6250 pseudo-long reads with a length of 500 kb. We then used Minimap2 (-cx map-hifi) to map them to the pre-polished CHM13 original assembly. Alignments with coverage ≤ 90% or identity ≤ 98% were removed, and 5868 pseudo-long reads with a total length of ∼ 2.93 Gb were retained for further analysis. We then mapped those pseudo-long reads to the original and polished CHM13 assemblies and calculated QV scores based on average alignment identity, using the formula $-10\times \log_{10}\left(1-\mathrm{Alignment}\;\mathrm{match}\;\mathrm{length}/\mathrm{Alignment}\;\mathrm{block}\;\mathrm{length}\right)$. We found that the QV of the RM-polished assembly decreased (Table 1). Moreover, the proportion of reads with 100% mapping identity reduced by 2.2% after RM polishing, and 47.09% of the reads had lower identity. In contrast, NextPolish2 slightly increased the proportion of reads with 100% mapping identity from 9.48% to 9.58% and only 14% of reads decreased in mapping identity after genome polishing (Table S5).

### NextPolish2 requires less computational resource than RM

On running time, NextPolish2 achieved error corrections significantly faster than RM ∼ 1–5 times and ∼ 10 times faster for the real and simulated datasets, respectively (Table 1). The betterment of the simulated dataset might be owing to the mentioned heterozygous issue. Moreover, NextPolish2 required less memory for the real datasets.

### Limitations of QV evaluation

Although QV is widely used to estimate the base-level accuracy of genome assemblies, we found that it cannot fully reflect the level of genome accuracy due to limitations in its estimation. This is because QV simply calculates how many *K*-mers in a *de novo* assembly are also found in unassembled high-accuracy reads. Briefly, two typical situations will increase the QV estimation but introduce unwanted assembly errors. (1) In a scenario of heterozygous *K*-mers, if the polishing tool changes an “incorrect” *K*-mer to a “correct” *K*-mer which is merely more abundant but derived from another haplotype, it increases QV but introduces haplotype switch errors (Figure S3A). (2) In the case of genome repeats that generate a varied number of reads, the correct *K*-mer may be replaced by an incorrect *K*-mer because it is not as well covered by the short-read set as the incorrect *K*-mer derived from other repeats (Figure S3B). This increases QV but leads to wrong assemblies for genome repeats. For example, RM polishing has introduced assembly errors in about 80.54% of TEs in the simulated *A*. *thaliana* genome, but the QV of these TEs showed an increase from 56.86 to 62.00 (Table S4). Additionally, the QV reported by Merqury of the RM-polished CHM13 assembly increased, but its alignment identity (QV) with the reference sequence (the CHM13 T2T genome) decreased (Table 1).

Another limitation of QV estimation by tools like Merqury is that it generates various QV scores for an identical assembly when using different coverages of short reads. For example, it showed that the QV of the same *A*. *thaliana* assembly (Col-XJTU), a score should be fixed but increased with an increased coverage of the input short-read data (Figure S4). Besides, we found that the polished genomes still contain errors, most of which were not covered by Illumina short reads or showed high inconsistencies among the mapped short reads (Figures S5 and S6). For example, 34.52% erroneous *K*-mers defined by Merqury in the polished *A*. *thaliana* assembly were in genome regions of mapping depth ≤ 3 when the average depth of input data was 104.82, and the reads spanning these erroneous *K*-mers only have an identity of 98.31%. Similarly, 76.29% erroneous *K*-mers in the polished HG002 assembly (child) were in genome regions of mapping depth ≤ 3 when the average depth of input data was 32.75, and the reads spanning these erroneous *K*-mers only have an identity of 95.37%.

Additionally, we found that the genome polishing tools designed for long noisy reads, such as Racon and NextPolish, introduced more errors than that they corrected (Table 1) and therefore are not recommended for error correction of genomes assembled using HiFi long reads.

## Discussion

NextPolish2 is a fast open-source polishing tool specifically developed for correcting errors in genomes assembled from HiFi long reads. It is an upgraded version of NextPolish and can additionally work on genomes assembled from noisy long reads, as well as those gap regions that are filled with sequences produced from ONT ultra-long reads in T2T genomes.

The performance of NextPolish2 relies heavily on short reads to check whether a *K*-mer contains errors. Therefore, we encourage users to use polymerase chain reaction (PCR)-free libraries and high-coverage short reads to minimize uncorrected errors caused by biases inherent in short-read sequencing technologies, especially for T2T genome projects that pursue extremely high-quality genome assemblies.

## Code availability

NextPolish2 is implemented in Rust. The source code as well as results of the benchmark tests is freely available from https://github.com/Nextomics/NextPolish2 and https://ngdc.cncb.ac.cn/biocode/tools/BT007383.

## CRediT author statement

**Jiang Hu:** Methodology, Software, Writing – original draft. **Zhuo Wang:** Methodology, Validation. **Fan Liang:** Validation. **Shan-Lin Liu:** Writing – review & editing. **Kai Ye:** Supervision. **De-Peng Wang:** Project administration. All authors have read and approved the final manuscript.

## Supplementary material

Supplementary material is available at *Genomics, Proteomics & Bioinformatics* online (https://doi.org/10.1093/gpbjnl/qzad009).

## Competing interests

De-Peng Wang is chief executive officer of GrandOmics Biosciences. Jiang Hu, Zhuo Wang, and Fan Liang are employees in GrandOmics Biosciences. All the other authors have declared no competing interests.

## Supplementary Material

qzad009_Supplementary_Data
