# Supplementary material for: NextPolish2: A Repeat-aware Polishing Tool for Genomes Assembled Using HiFi Long Reads
Source: Genomics Proteomics Bioinformatics. 2024 Jan 4;22(1):qzad009. doi: 10.1093/gpbjnl/qzad009 (PMC12016036; doi:10.1093/gpbjnl/qzad009)
Supplement: qzad009_Supplementary_Data [file qzad009_supplementary_data.zip › Supplementary material - Captions.docx]

## Supplementary material

**Figure S1 Integrative genomics viewer screenshot of a deletion error in the reference genome**

HiFi, high-fidelity.

**Figure S2 Integrative genomics viewer screenshot of a SNV error in the reference genome**

SNV, single nucleotide variants.

**Figure S3 The schematic of two overcorrection cases introduced by genome polishing**

**A.** The mapped HiFi reads show that there are three haplotypes or repeat copies (h1, h2, and h3 are represented by grey, pink, and green, respectively) in this region. The raw genome (reference) is corrected as a mixed haplotype, which does not actually exist in the HiFi data. However, this correction leads to an improved QV, as erroneous K-mers (K-merx) are corrected as K-mer2 and K-mer3 that present in short reads. **B.** The correct K-mer2 shown in green in the raw genome (reference) is corrected as an erroneous K-merN because K-mer2 is missing in short reads due to sequencing biases or insufficient coverage, and K-merN is present in short reads but from another region. This correction can also lead to an improved QV. QV, quality value.

**Figure S4 The QV of the *A*. *thaliana* (Col-XJTU) assembly evaluated by Merqury increases as the input short-read data increases**

*A*. *thaliana*, *Arabidopsis thaliana*.

**Figure S5 Integrative genomics viewer screenshot of an error region defined by Merqury with K-mers that are missing from Illumina reads**

**Figure S6 Integrative genomics viewer screenshot of an error region defined by Merqury that are inconsistent with Illumina data**

Some mapped reads without SNPs/InDels do not span the K-mer interval. SNPs, single nucleotide polymorphisms; InDels, insertions and deletions.

**Table S1 Statistical information of the datasets used in this study**

**Table S2 Statistics of genome polishing results on three additional datasets**

**Table S3 Statistics of potential homozygous K-mer changes on all datasets**

**Table S4 Accuracy of transposable elements in the simulated *A*. *thaliana* genome before and after polishing**

**Table S5 Accuracy of pseudo-long reads in the *H*. *sapiens* (CHM13) genome before and after polishing**
