# Supplementary material for: NextPolish2: A Repeat-aware Polishing Tool for Genomes Assembled Using HiFi Long Reads
Source: Genomics Proteomics Bioinformatics. 2024 Jan 4;22(1):qzad009. doi: 10.1093/gpbjnl/qzad009 (PMC12016036; doi:10.1093/gpbjnl/qzad009)
Supplement: qzad009_Supplementary_Data [file qzad009_supplementary_data.zip › Table S1-done.docx]

**Table S1 Statistical information of the datasets used in this study**

| **Source** | **Read type** | **Bases (bp)** | **Average reads length (bp)** | **Base coverage** |
| --- | --- | --- | --- | --- |
| *A*. *thaliana* (simulated data) | Illumina | 13,207,830,000 | 150 | 100.00 |
|  | HiFi | 8,343,317,687 | 12,956 | 63.17 |
| *A*. *thaliana* | Illumina | 13,696,431,300 | 150 | 104.82 |
|  | HiFi | 4,696,718,342 | 15,098 | 35.95 |
| *H*. *sapiens* (HG002) | Illumina | 100,892,786,960 | 148 | 32.75 |
|  | HiFi | 110,549,151,396 | 14,971 | 35.88 |
| *H*. *sapiens* (HG003) | Illumina | 101,741,039,192 | 148 | 33.02 |
| *H*. *sapiens* (HG004) | Illumina | 101,309,565,616 | 148 | 32.88 |
| *H*. *sapiens* (CHM13) | Illumina | 409,858,305,000 | 250 | 131.48 |
|  | HiFi | 100,369,168,661 | 18,028 | 32.20 |

*Note*: HiFi, high-fidelity.
