# Supplementary material for: NextPolish2: A Repeat-aware Polishing Tool for Genomes Assembled Using HiFi Long Reads
Source: Genomics Proteomics Bioinformatics. 2024 Jan 4;22(1):qzad009. doi: 10.1093/gpbjnl/qzad009 (PMC12016036; doi:10.1093/gpbjnl/qzad009)
Supplement: qzad009_Supplementary_Data [file qzad009_supplementary_data.zip › Table S2-done.docx]

**Table S2 Statistics of genome polishing results on three additional datasets**

| **Source** | **Software** | **QV** | **Changed K-mers** | **Potential overcorrection K-mers** |
| --- | --- | --- | --- | --- |
| *A*. *thaliana* (134 M) Col-XJTU (CNCB-NGDC:PRJCA005809) | hifiasm | 62.90 |  |  |
|  | Racon + Merfin | **64.91** | 7061 | 45 |
|  | NextPolish2 | 64.54 | **3548** | **0** |
| *Oryza sativa* J4155S (407 M) (CNCB-NGDC:PROJCA008812) | hifiasm | 47.42 |  |  |
|  | Racon + Merfin | 49.51 | 40,530 | 1113 |
|  | NextPolish2 | **49.59** | **38,289** | **72** |
| *Monopterus albus* (862 M) (CNCB-NGDC:PRJCA008725) | hifiasm | 52.45 |  |  |
|  | Racon + Merfin | **57.47** | 11,494,206 | 2287 |
|  | NextPolish2 | 56.20 | **850,030** | **392** |

*Note*: The *A*. *thaliana* dataset used here is different from Table 1. The HiFi data in Table 1 was downsampled to 30×, and the genome was assembled from this 30× data. This table used all 171× HIFi data and the genome was download from the web. The best value for each metrics is indicated in bold.
