# Supplementary material for: NextPolish2: A Repeat-aware Polishing Tool for Genomes Assembled Using HiFi Long Reads
Source: Genomics Proteomics Bioinformatics. 2024 Jan 4;22(1):qzad009. doi: 10.1093/gpbjnl/qzad009 (PMC12016036; doi:10.1093/gpbjnl/qzad009)
Supplement: qzad009_Supplementary_Data [file qzad009_supplementary_data.zip › Table S3-done.docx]

**Table S3 Statistics of potential homozygous K-mer changes on all datasets**

| **Table** | **Source** | **Software** | **Homozygous K-mer (#)** |
| --- | --- | --- | --- |
| Table 1 | *A*. *thaliana* (simulated data, primary contigs) | Racon + Merfin | 5,783,074 |
|  |  | NextPolish2 | **1648** |
|  | *A*. *thaliana* (Col-XJTU, primary contigs) | Racon + Merfin | 2210 |
|  |  | NextPolish2 | **93** |
|  | *H*. *sapiens* (HG002, primary contigs) | Racon + Merfin | 231,128 |
|  |  | NextPolish2 | **918** |
|  | *H*. *sapiens* (HG002, paternal contigs) | Racon + Merfin | 185,034 |
|  |  | NextPolish2 | **60,463** |
|  | *H*. *sapiens* (HG002, maternal contigs) | Racon + Merfin | 152,712 |
|  |  | NextPolish2 | **10,081** |
|  | *H*. *sapiens* (CHM13, primary contigs) | Racon + Merfin | 275,495 |
|  |  | NextPolish2 | **1843** |
| Table S2 | *A*. *thaliana* (134 M) Col-XJTUa (CNCB-NGDC:PRJCA005809) | Racon + Merfin | 1681 |
|  |  | NextPolish2 | **26** |
|  | *Oryza sativa* J4155S (407 M) (CNCB-NGDC:PROJCA008812) | Racon + Merfin | 2398 |
|  |  | NextPolish2 | **943** |
|  | *Monopterus albus* (862 M) (CNCB-NGDC:PRJCA008725) | Racon + Merfin | 222,269 |
|  |  | NextPolish2 | **23,639** |

*Note*: Homozygous K-mers are defined as K-mers that exist in primary assembly, but are removed in polishing and the depth (multiplicity) of K-mers ≥ P * 0.8 and ≤ P * 1.2, where P is peak depth of K-mers derived from Illumina short reads. The best value for each metrics is indicated in bold.
