# Supplementary material for: NextPolish2: A Repeat-aware Polishing Tool for Genomes Assembled Using HiFi Long Reads
Source: Genomics Proteomics Bioinformatics. 2024 Jan 4;22(1):qzad009. doi: 10.1093/gpbjnl/qzad009 (PMC12016036; doi:10.1093/gpbjnl/qzad009)
Supplement: qzad009_Supplementary_Data [file qzad009_supplementary_data.zip › Table S4-done.docx]

**Table S4 Accuracy of transposable elements in the simulated *A. thaliana* genome before and after polishing**

| **Source** | **Software** | **A total of 149 transposable elements** | | | |
| --- | --- | --- | --- | --- | --- |
|  |  | **QV (all)** | **100% mapping identity (%)** | **Lower identity after polishing** | |
|  |  |  |  | **Percent (%)** | **QV (lower)** |
| *A*. *thaliana* (simulated data, primary contigs) | hifiasm (primary) | 56.58 | 68.46 |  | 56.86 |
|  | Racon + Merfin | 61.64 | 12.75 | 80.54 | 62.00 |
|  | NextPolish2 | **inf** | **91.95** | **0.00** | **inf** |

*Note*: QV (all) refers to the QV evaluated by Merqury from all 149 transposons. QV (lower) refers to the QV evaluated by Merqury from 120 transposons with lower identity after polishing by RM. hifiasm (primary) refers to the primary hifiasm assembly. The identity was defined by Minimap2 and only the primary alignments of each transposable element were used for evaluation. “inf” is the result of a numerical calculation that is mathematically infinite. The best value for each metrics is indicated in bold. RM, Racon + Merfin.
