# Supplementary material for: NextPolish2: A Repeat-aware Polishing Tool for Genomes Assembled Using HiFi Long Reads
Source: Genomics Proteomics Bioinformatics. 2024 Jan 4;22(1):qzad009. doi: 10.1093/gpbjnl/qzad009 (PMC12016036; doi:10.1093/gpbjnl/qzad009)
Supplement: qzad009_Supplementary_Data [file qzad009_supplementary_data.zip › Table S5-done.docx]

**Table S5 Accuracy of pseudo-long reads in the *H. sapiens* (CHM13) genome before and after polishing**

| **Source** | **Software** | **A total of 5868 pseudo–long reads** | | | |
| --- | --- | --- | --- | --- | --- |
|  |  | **QV (all)** | **100% mapping identity (%)** | **Lower identity after polishing** | |
|  |  |  |  | **Percent (%)** | **QV (lower)** |
| *H*. *sapiens* (CHM13, primary contigs) | hifiasm (primary) | 67.02 | 9.48 |  | 68.09 |
|  | Racon + Merfin | 69.46 | 7.28 | 47.09 | 68.62 |
|  | NextPolish2 | **69.54** | **9.58** | **14.00** | **69.64** |

*Note*: QV (all) refers to the QV evaluated by Merqury from regions in each assembly mapped by all 5868 pseudo-long reads. QV (lower) refers to the QV evaluated by Merqury from regions in each assembly mapped by 3175 pseudo-long reads with lower identity after polishing by RM or NextPolish2. hifiasm (primary) refers to the primary hifiasm assembly. The identity was defined by Minimap2 and only the primary alignments of each pseudo-long reads were used for evaluation. The best value for each metrics is indicated in bold.
