# Supplementary figures and images for: NextPolish2: A Repeat-aware Polishing Tool for Genomes Assembled Using HiFi Long Reads
Source: Genomics Proteomics Bioinformatics. 2024 Jan 4;22(1):qzad009. doi: 10.1093/gpbjnl/qzad009 (PMC12016036; doi:10.1093/gpbjnl/qzad009)

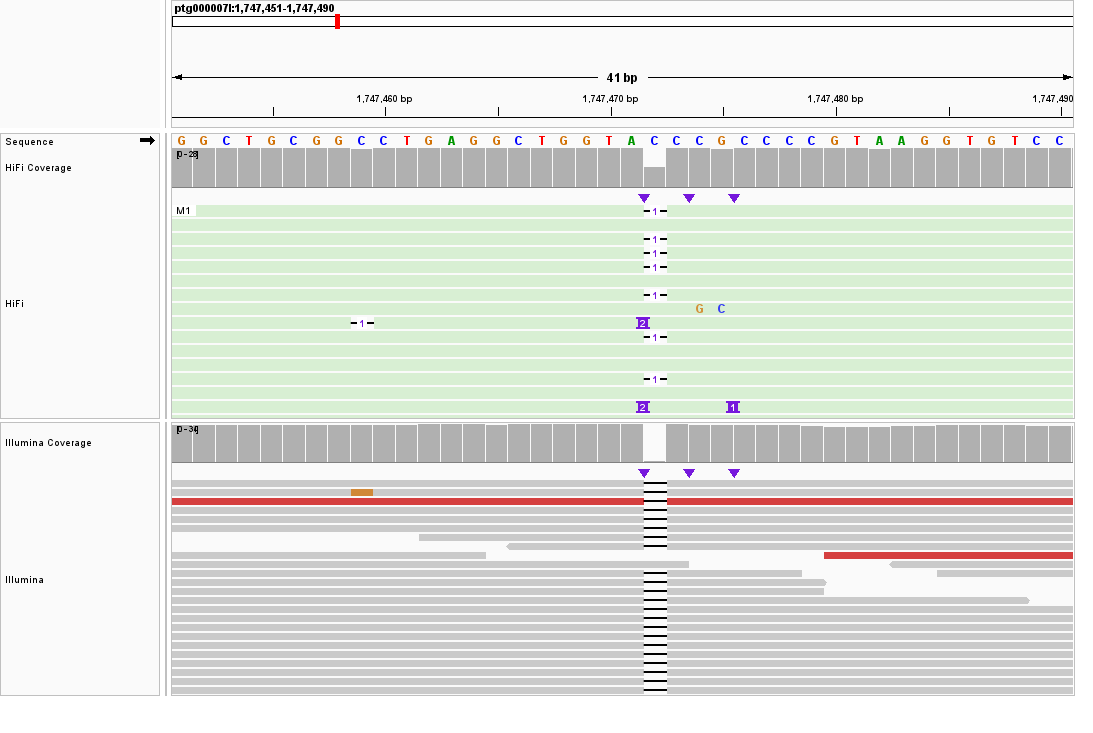

Supplement: qzad009_Supplementary_Data [file qzad009_supplementary_data.zip › Figure S1.png]

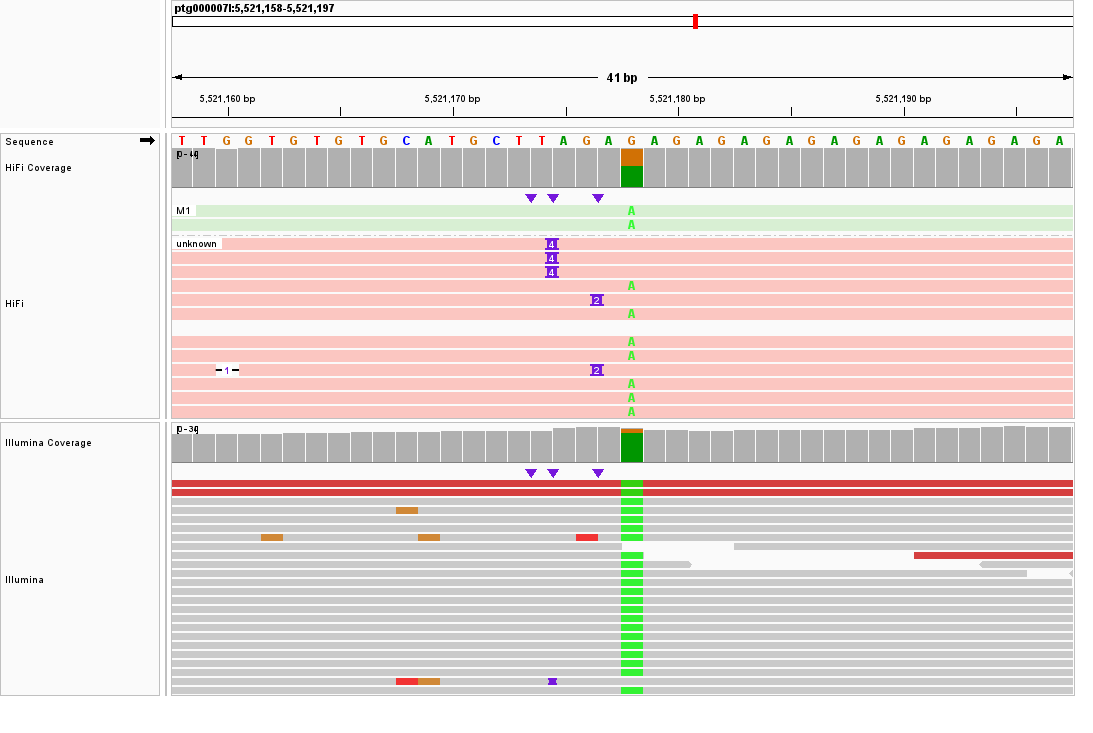

Supplement: qzad009_Supplementary_Data [file qzad009_supplementary_data.zip › Figure S2.png]

**A**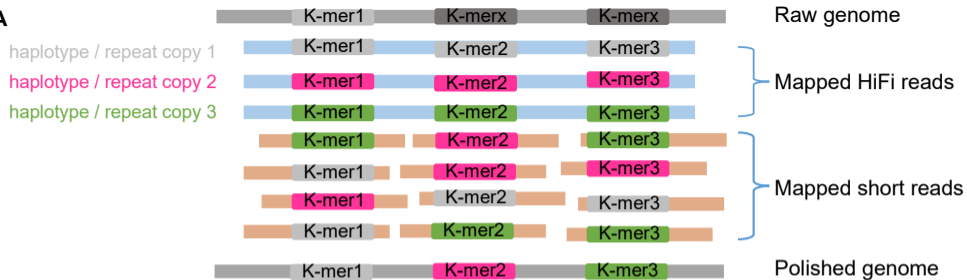**B**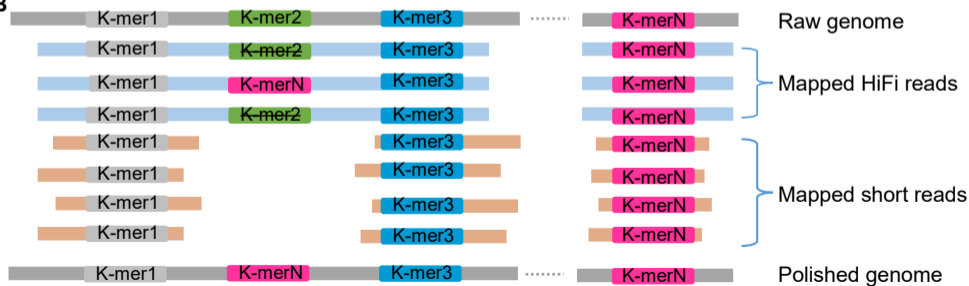

Supplement: qzad009_Supplementary_Data [file qzad009_supplementary_data.zip › Figure S3.pdf]

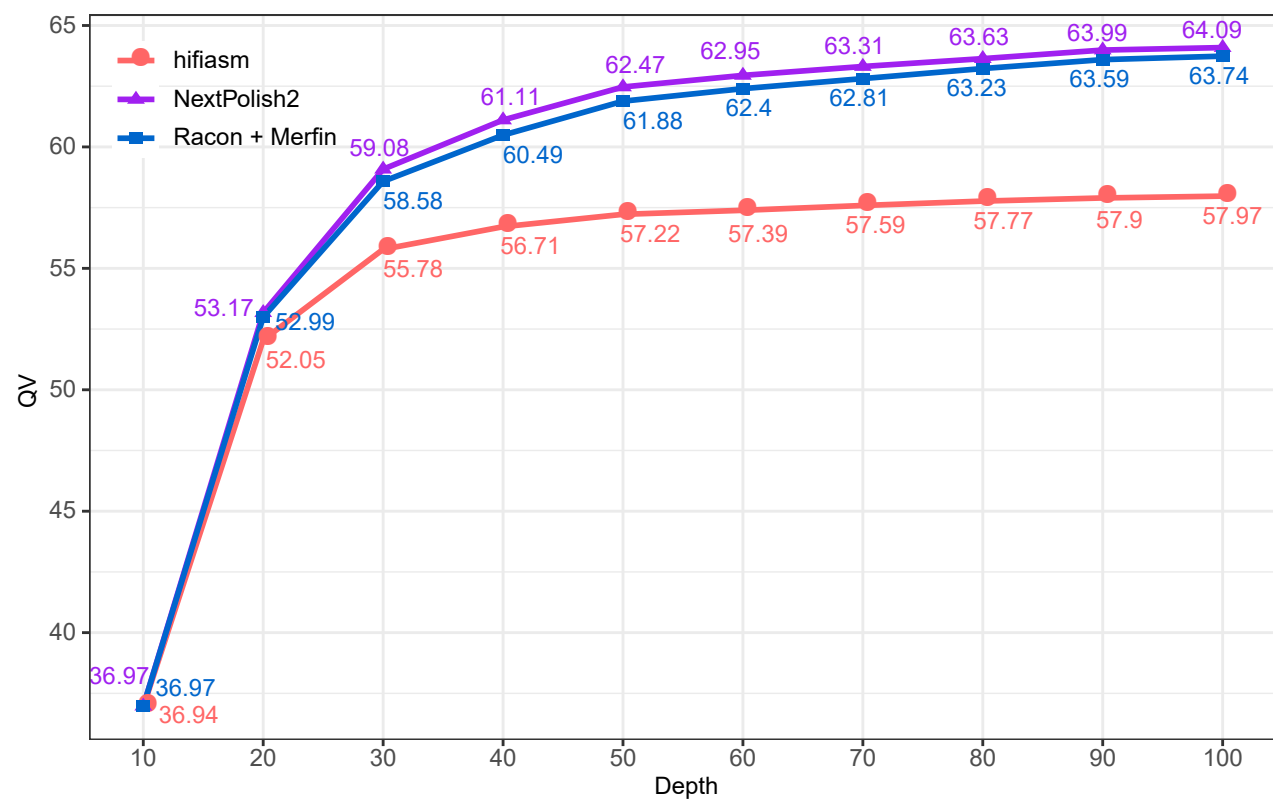

Supplement: qzad009_Supplementary_Data [file qzad009_supplementary_data.zip › Figure S4.pdf]

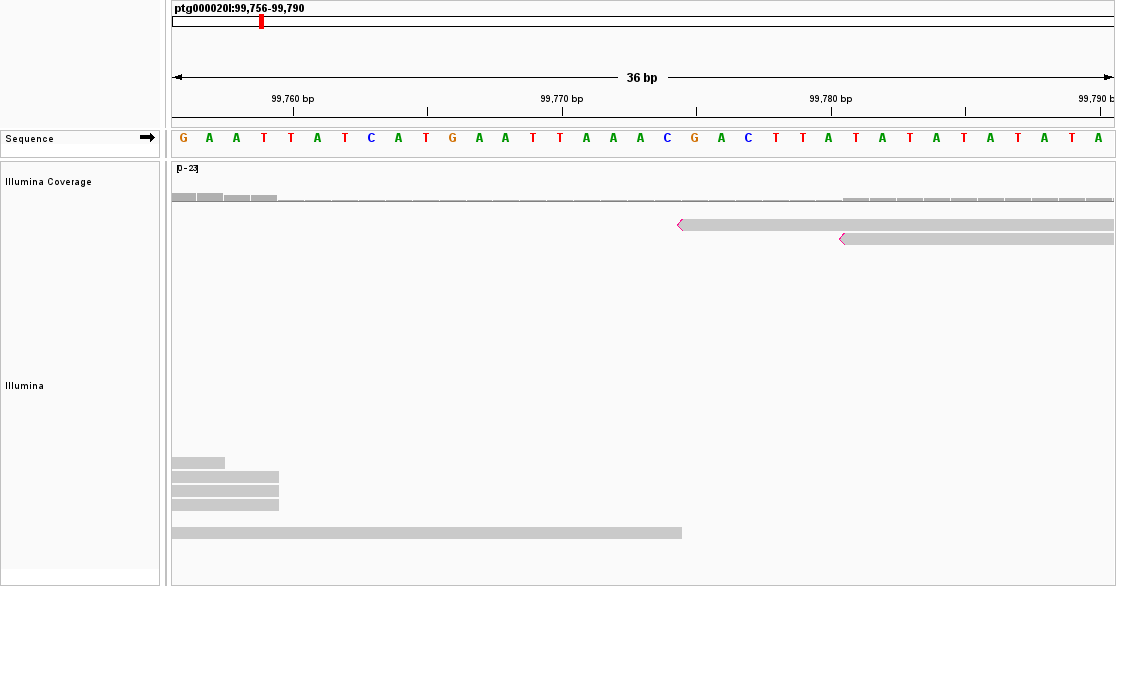

Supplement: qzad009_Supplementary_Data [file qzad009_supplementary_data.zip › Figure S5.png]

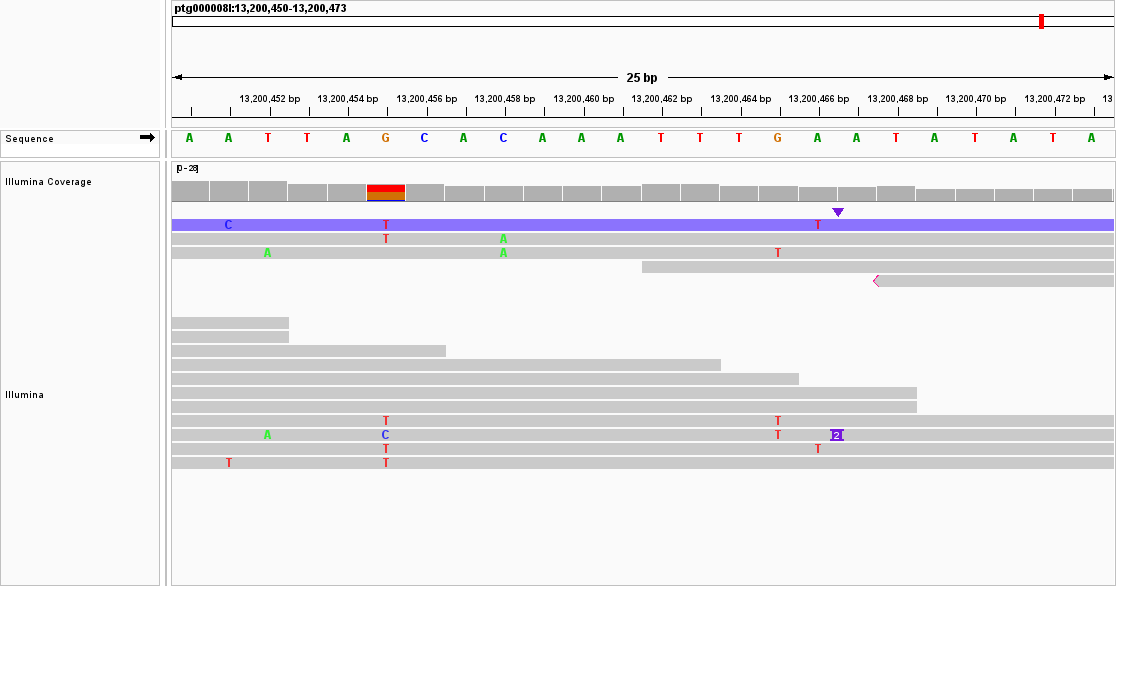

Supplement: qzad009_Supplementary_Data [file qzad009_supplementary_data.zip › Figure S6.png]
